# Chemical Characterization of Gallstones: An Approach to Explore the Aetiopathogenesis of Gallstone Disease in Sri Lanka

**DOI:** 10.1371/journal.pone.0121537

**Published:** 2015-04-08

**Authors:** Harshi Weerakoon, Ayanthi Navaratne, Shirani Ranasinghe, Ramaiah Sivakanesan, Kuda Banda Galketiya, Shanthini Rosairo

**Affiliations:** 1 Department of Biochemistry, Faculty of Medicine and Allied Sciences, Rajarata University of Sri Lanka, Saliyapura, Sri Lanka; 2 Department of Chemistry, Faculty of Science, University of Peradeniya, Peradeniya, Sri Lanka; 3 Department of Biochemistry, Faculty of Medicine, University of Peradeniya, Peradeniya, Sri Lanka; 4 Department of Surgery, Faculty of Medicine, University of Peradeniya, Peradeniya, Sri Lanka; 5 Department of Radiology, Faculty of Medicine, University of Peradeniya, Peradeniya, Sri Lanka; 6 Post Graduate Institute of Science, University of Peradeniya, Peradeniya, Sri Lanka; SGPGI, INDIA

## Abstract

**Introduction:**

Records on gallstones and associated ailments in Sri Lankan community are scarce, despite frequent detection of gallstone disease. Identification of the chemical composition of gallstones in the local setting is important in defining aetiopathogenic factors which in turn are useful in implementing therapeutic and preventive strategies. This study aimed to describe the chemical composition of gallstones and the socio-demographic factors of a cohort of Sri Lankan patients with gallstone disease.

**Materials and Methods:**

Data on clinical and socio-demographic factors, and gallstones removed at surgery were collected from patients with cholelithiasis admitted to Teaching Hospital, Peradeniya, Sri Lanka from May 2011 to December 2012. External and cross sectional morphological features of gallstones were recorded by naked eye observation. Compositional analysis was carried out by Fourier Transform Infrared Spectroscopy, X - ray Powder Diffraction, and Atomic Absorption Spectrophotometry. Scanning Electron Microscopy was used to identify the microstructure of gallstones.

**Results:**

Data of 102 patients were analyzed. Of them majority (*n* = 77, 76%) were females with a female: male ratio of 3:1. Mean age of the study group was 46.1±11.6 years. All the patients had primary gallbladder stones. According to the physical and chemical analysis, majority (*n* = 54, 53%) were pigment gallstones followed by mixed cholesterol gallstones (*n* = 38, 37%). Only 10 (9%) had pure cholesterol gallstones. Calcium bilirubinate, calcium carbonate and calcium phosphate were the commonest calcium salts identified in pigment gallstones and core of mixed cholesterol gallstones.

**Conclusion:**

Presence of a pigment nidus in gallstones is a common feature in majority of Sri Lankan patients denoting the possible role of elevated unconjugated bilirubin in bile on the pathogenesis of GS. Hence it is imperative to explore this further to understand the aetiopathogenesis of GS among Sri Lankans.

## Introduction

Gallstones (GS) are formed in the gallbladder (GB) and bile duct, from the constituents of bile [1]. Cholesterol and calcium bilirubinate are the main chemical compounds present in GS and their precipitation in bile is induced by multiple aetiological factors. Therefore, chemical composition of GS would indicate the factors involved in the process of development of these GS. In GS, cholesterol is deposited mainly due to the supersaturation of cholesterol in bile [2], while calcium bilirubinate precipitation occurs due to defective bilirubin conjugation [1, 3]. Apart from these, calcium carbonate, calcium phosphate and calcium fatty acids (such as palmitate and stearate) are found in GS as minor compounds [4]. A wide range of elements including heavy metals are also detected in varying concentrations in different types of GS [5–8] and these trace minerals are suspected to be involved in the development of pigment GS as well [5].

Depending on their major composition, GS are commonly divided into two main categories as cholesterol and pigment GS [1]. Cholesterol is the main chemical compound identified in cholesterol GS, whereas calcium bilirubinate is present as the main chemical compound in pigment GS [1]. In the Japanese classification system, these two main types are further subdivided based on the cross sectional morphology [4]. In this classification, cholesterol stones are subgrouped as pure, mixed and combination stones. Pigment stones are further classified as black and calcium bilirubinate (brown) stones [4].

The prevalence and the chemical composition of GS vary from population to population [9]. This indicates the involvement of multiple aetiological factors in the pathogenesis of GS [9]. Therefore investigation of the chemical composition of GS is primarily important to recognize the aetiological factors for GS disease in a given community. GS disease has not been adequately investigated in Sri Lankan population despite its high prevalence according to the hospital records. Apart from the identification of bacterial colonies in bile of Sri Lankan patients with GS disease [10], studies to identify the mechanism of formation of GS have not yet been carried out. Moreover, a high prevalence (10–12%) of GS disease and its complications (eg; GS carcinoma) are reported from India, the closest neighbor of Sri Lanka [11]. Based on these facts, an extensive description on GS disease among Sri Lankans can be considered imperative. Further, identification of chemical composition of GS among Sri Lankans would be an important gateway to explore the aetiopathogenesis of GS disease.

Fourier Transform Infrared Spectroscopy (FTIR) is the most widely used technique [12–15] in the analysis of chemical composition of GS, as it is accurate, less time consuming and cost effective. Thus it is the main technique used in the large scale analysis of GS samples [13]. Moreover the chemical analysis of GS by X—ray Powder Diffraction (XRD) [16–17] and colorimetric assays [18] are also available. XRD is used to describe the crystalline composition of GS, while microstructure of the GS is identified by Scanning Electron Microscopy (SEM) [12, 19] and, currently it is considered as the best method of describing the microstructure of GS. Elemental composition of these GS is commonly analyzed using Atomic Absorption Spectrophotometry (AAS) and Particle Induced X-Ray Emission [7, 8]. Currently the conventional GS classification has also been modified with the findings of the chemical composition of GS with these advanced analytical techniques [12].

The objectives of this study were to describe the chemical composition of GS in a cohort of patients with GS disease, admitted to Teaching Hospital, Peradeniya, Sri Lanka and their socio-demographic factors to understand the aetiopathogenesis of the condition.

## Materials and Methods

### Socio-demographic data and GS sample collection

The study was conducted at Teaching Hospital, Peradeniya, Sri Lanka during May 2011 to December 2012. Ethical clearance for the study was obtained from the Ethics Review Committee, Faculty of Medicine, University of Peradeniya, Sri Lanka. Upon receiving the informed written consent, socio-demographic data were recorded from the adult patients residing in Kandy district for more than 5 years, who were admitted for GS removal surgeries due to symptomatic GS.

GS removed at the surgery were collected and washed immediately with running water and were air dried. Samples were then washed with distilled water and deionized water before dried in an electric oven at 60°C to achieve a constant weight. All GS recovered from a single patient was considered as one sample. The external morphological features of the stones were recorded by naked eye observation and the largest stone from each sample was cut into two halves to record the cross sectional appearance. GS were initially categorized based on the cross sectional appearance, as per the Japanese classification [4]. Powdered GS samples were used for the FTIR, XRD and AAS analysis.

### Analysis of samples by FTIR

In FTIR analysis, 5 mg of GS powder was mixed with 100 mg of potassium bromide (KBr) to prepare pellets with a diameter of 13 mm. One sample from GS with homogenous morphology and multiple samples representing distinct layers of GS with heterogeneous morphology were taken for the FTIR analysis. The measurements were taken using Shimadzu IR Prestige 21 instrument at mid frequency range (4000–400 cm^–1^) at 4 cm^–1^ resolution. FTIR spectra of cholesterol, bilirubin, calcium carbonate, calcium phosphate and palmitic acid were taken from the standard compounds (90% pure) purchased from Sigma Aldrich, Germany.

Chemical composition of GS in each type of GS was identified comparing the FTIR patterns of the samples with that of the standards. Five samples from each group were randomly selected to carry out XRD studies to confirm FTIR results.

### Analysis of samples by XRD

Siemens X-ray diffractometer (Model- D50000) with Cu-K X-ray beam (wave length of the radiation -1.54056 Ǻ) was used for the XRD analysis. According to the Bragg equation; **n λ =** 2**d** sin θ (n—integer, λ—wavelength of the radiation, d—inter-planar distance, θ—diffraction angle), d- spacing values were calculated from the two theta degree values obtained by XRD. Diffraction patterns of standard chemical compounds were taken and the identification of crystalline compounds present in each GS sample was done comparing the d-spacing values obtained for the GS samples with that of the standard compounds.

### Analysis of GS by SEM

Three GS with distinct cross sectional morphologies were subjected to SEM analysis. Each stone was sliced into pieces with a breadth of about 3–5 mm, to include morphologically different layers. Using an electro-conductive adhesive, each sample was fixed on the sample table where one side was secured to the surface and the other was placed facing the opposite direction and the surface to be analyzed was polished. After drying at 60°C overnight, the samples were coated with gold and observed using a SEM (Hitachi SU 6600) and photographs were taken to identify the microstructures of different types of GS. Elemental composition of these GS was taken by Energy Dispersive X-ray Spectroscopy (EDS).

### Estimation of Pb^2+^ and Cd^2+^ concentrations in GS

GS samples which are more than 500 mg in weight were used for the heavy metal analysis, in which 500 mg powder samples were digested using dry ashing technique (pigment GS *n* = 20, cholesterol GS *n* = 14). Samples were ashed in a tube furnace (Carbolite CTF12/100/900) at 200°C for 1 hour, 450°C for 2 hours and 500°C for 5 hours. Ashed samples were then dissolved in 1 cm^-3^ of 1 moldL^-3^ Nitric acid. Each sample was filtered into 25 cm^-3^ volumetric flasks and made up to 25 cm^-3^ with deionized water. Standard samples for Pb^**2+**^ and Cd^**2+**^ were prepared using standard solutions purchased from Fluka Chemica, Switzerland.

## Results

### Socio-demographic features of the subjects

A total of 102 patients, who fulfilled the inclusion criteria, underwent cholecystectomy surgeries during the study period. Of them 77 (76%) were females and 25 (24%) were males, resulting in a female to male ratio of 3:1. The age of the study group ranged from 27 to 82 years with a mean of 46.1±11.6 years. Mean ages of presentation with symptomatic GS showed a significant difference (p = 0.004) between females and males with 44.1 ± 10.8 and 52.2 ± 12.0 years respectively.

Of the 102 patients, 82 (80%) had GS in GB while remaining 20 (20%) had GS both in GB and bile ducts. None had primary bile duct stones.

### Classification of GS by cross sectional morphology

**Fig 1:**
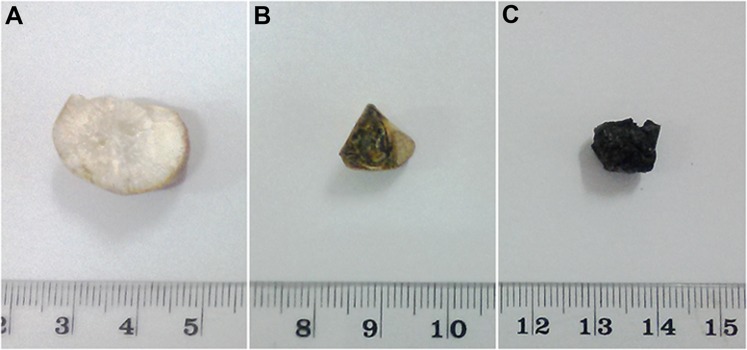
Cross sectional appearance of each type of gallstone. A—Pure cholesterol stone showing radially arranged cholesterol crystals from center to periphery. B—Mixed cholesterol stone showing cholesterol and pigment layers arranged alternatively in crescentric pattern. C—Pigment stone showing homogenously distributed pigment material.

Cross sectional patterns identified in the GS are shown in Fig 1. Of the total, 10 (9%) of the GS showed a radially distributed homogenous material (Fig 1A) and were categorized as pure cholesterol GS according to the Japanese classification system criteria [4]. GS which had crescentric layers of dark (black or brown) and light (pale white) colour material from center to periphery (Fig 1B) were named as mixed cholesterol GS (*n* = 38, 37%) [4]. Core of all the GS classified as mixed cholesterol was dark in colour. All the remaining GS (*n* = 54, 54%) which had homogenously distributed black material (Fig 1C) in their cross sections were categorized as pigment GS [4].

### Identification of chemical composition of GS by FTIR

**Fig 2:**
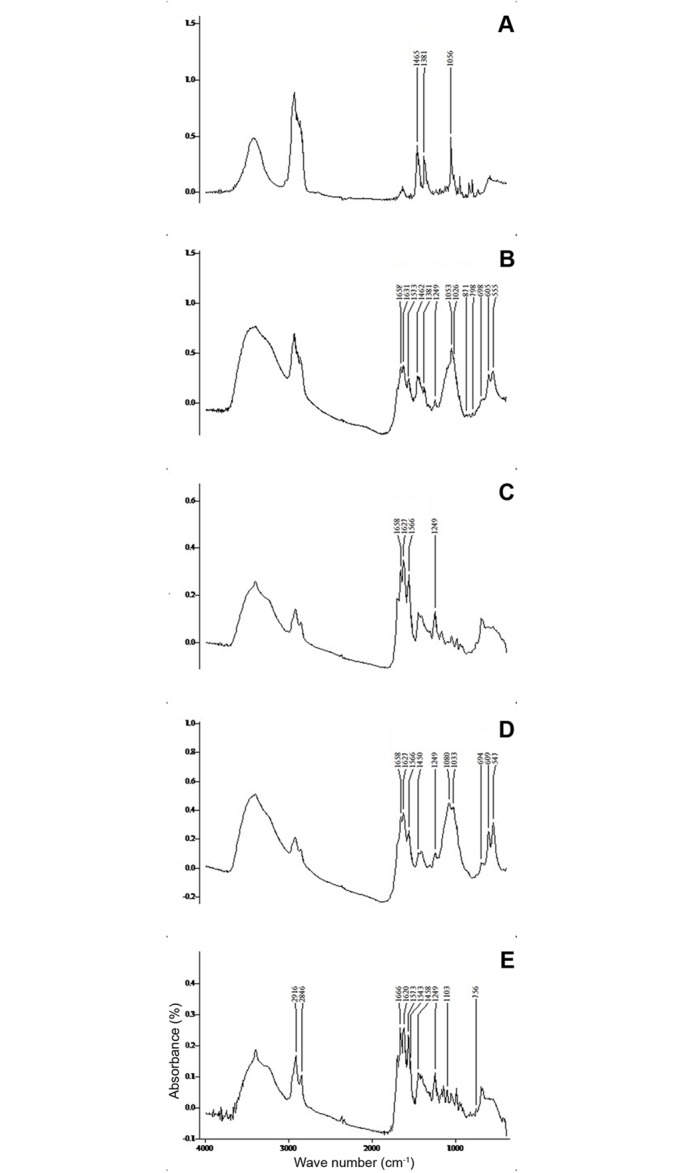
A typical FTIR spectrogram of five gallstone types. A—Pure cholesterol stone and pale areas of mixed cholesterol stone composed of cholesterol (1465, 1381, and 1056 cm^-1^). B—Pigmented areas of mixed cholesterol stone composed of calcium bilirubinate (1658, 1631, 1573 and 1249 cm^-1^), calcium carbonate (1462, 871 and 698 cm^-1^) and calcium phosphate (1026, 606 and 505 cm^-1^). C—Pigment stone composed of calcium bilirubinate (1658, 1627, 1566 and 1249 cm^-1^). D—Pigment stone composed of calcium bilirubinate (1658, 1627, 1566 and 1249 cm^-1^), calcium carbonate (1450, 694 1658, 1627, 1566 and 1249 cm^-1^) and calcium phosphate (1080, 1033, 609 and 547 cm^-1^). E—Pigment stone composed of calcium bilirubinate (1666, 1620, 1573 and 1249 cm^-1^) and calcium palmitate (2916, 2846, 1543, 1103 and 756 cm^-1^).

The chemical compositions of different types GS as revealed by FTIR are shown in Table 1 and the patterns of FTIR spectra obtained for different types of GS are given in Fig 2. According to the analysis, majority of GS (*n* = 69, 68%) were composed of multiple chemical compounds, while only 33 (32%) of GS were composed only of single compound. Calcium bilirubinate, calcium carbonate and calcium phosphate are the frequently identified calcium salts in the GS samples whereas calcium palmitate was detected only in 6 (6%) pigment GS (Fig 2E). Further the chemical analysis of the core of mixed cholesterol GS (Fig 2B), which had heterogeneous morphology in cross section, revealed the presence of calcium bilirubinate, calcium carbonate and calcium phosphate.

**Table 1:** Chemical composition of gallstones as revealed by FTIR.

****Type of GS****	****Chemical composition****	****Frequency*****n*****(%)****
Pure cholesterol	Cholesterol	10 (09)
Mixed cholesterol	Cholesterol, calcium bilirubinate, calcium carbonate, calcium phosphate	38 (37)
Pigment	Calcium bilirubinate	23 (23)
	Calcium bilirubinate, calcium carbonate, calcium phosphate	25 (25)
	Calcium bilirubinate and calcium palmitate	06 (06)

### Identification of crystalline composition of GS by XRD

**Fig 3:**
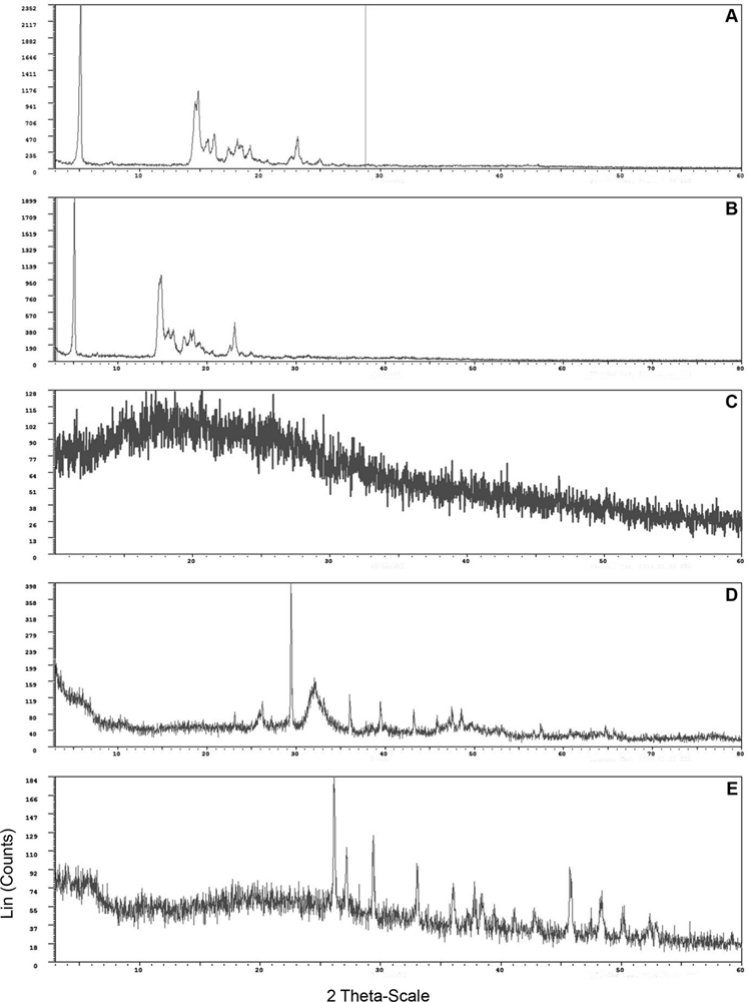
A typical X—Ray diffractogram of five gallstone types. A—Pure cholesterol stone. B—Mixed cholesterol stone. C—Pigment stone composed of calcium bilirubinate. D—Pigment stone composed of calcium bilirubinate, calcium carbonate and calcium phosphate. E—Pigment stone composed of calcium bilirubinate and calcium palmitate.

Diffraction patterns obtained for different types of GS are given in Fig 3. XRD analysis of pure cholesterol GS gave d-spacing values (Fig 3A) compatible to that of the d-values obtained for cholesterol standard. These stones showed intense peaks at 5.90, 5.47, 4.89, 3.80 and 6.30 Å which confirms the presence of cholesterol. d-spacing values obtained in XRD for the mixed cholesterol GS (Fig 3B) further confirm the presence of cholesterol (17.30, 3.80, 5.84, 6.30 and 6.94 Å), calcium carbonate (3.84, 3.03, 1.87, 2.49 and 1.93 Å) and calcium phosphate (3.40, 3.37, 4.17, 1.94 and 2.70 Å) in crystalline form. In XRD analysis of pigment GS a diffraction pattern was obtained indicating the presence calcium bilirubinate, carbonate (3.03, 2.49, 2.28, 1.87 and 1.91 Å), phosphate (2.78, 2.70, 3.40, 1.94 and 3.37 Å) (Fig 3D) or palmitate (4.10, 3.67, 3.49, 3.01 and 2.48Å) (Fig 3E).

### Microstructure analysis of GS by SEM

**Fig 4:**
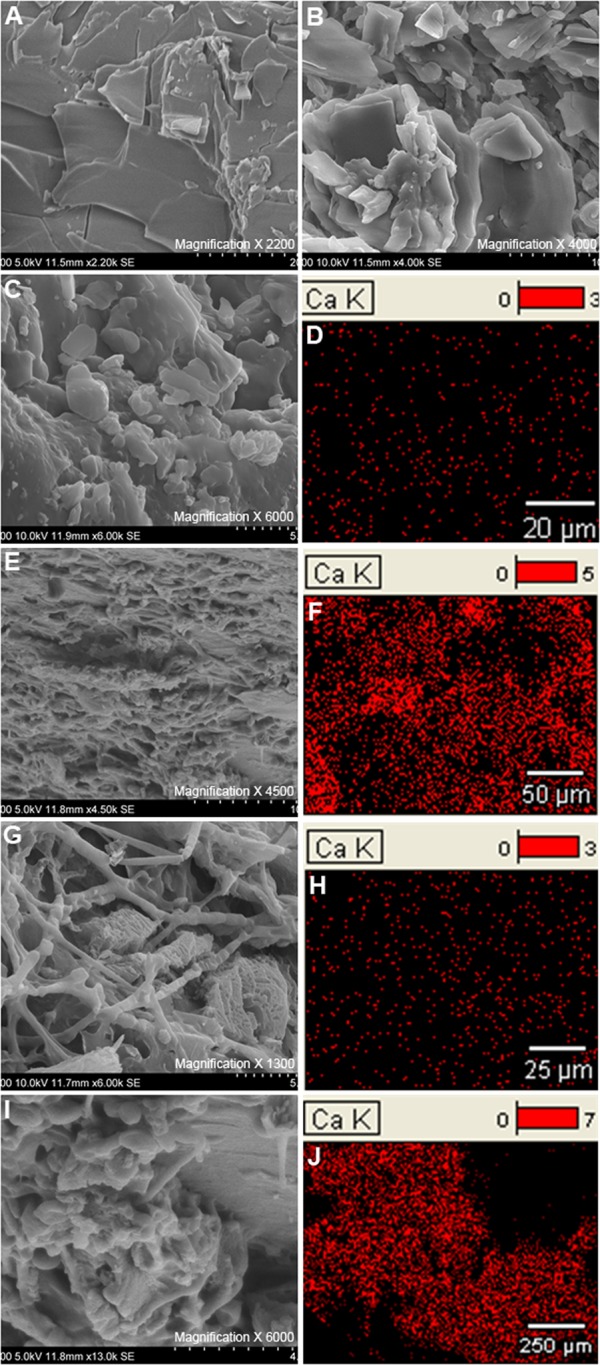
Microstructure and calcium distribution of different types of gallstones. A, B—Pure cholesterol stone showing plate and lamella shaped cholesterol crystals. C—Mixed cholesterol stone showing predominantly distributed lamella shaped cholesterol particles with scattered calcium bilirubinate particles. D—Calcium distribution of the mixed cholesterol GS. E- Pigment stone showing irregularly arranged calcium bilirubinate clumps. F—Calcium distribution of the pigment GS of an area composed of calcium bilirubinate in the pigment GS. G—Pigment stone showing irregularly arranged calcium bilirubinate particles and protein streaks. H—Calcium distribution of the pigment stone of an area composed of calcium bilirubinate and protein. I—Pigment stone showing bulbiform calcium phosphate crystals. J—Calcium distribution of the pigment stone of an area composed of calcium phosphate crystals.

SEM images and the calcium distribution obtained for three types of stones are shown in Fig 4 and the elemental composition obtained by EDS is summarized in Table 2. In pure cholesterol GS, cholesterol crystals appeared as tightly packed plate or lamella shaped structures (Fig 4A and 4B). Detection of carbon as the main element (> 99% of weight) by EDS followed by oxygen suggests the presence of cholesterol crystals. Detection of calcium in very minor percentage by weight and the absence of nitrogen and phosphorus confirms that this stone as a pure cholesterol GS. Similar to the pure cholesterol GS, plate and lamellar shaped structures suggestive of cholesterol were detected in the mixed cholesterol GS (Fig 4C). The irregular small particles seen in addition to cholesterol crystals (Fig 4C) were identified as calcium bilirubinate according to the EDS results (Fig 4D). The irregular small particles (Fig 4E) seen in the pigment stone were identified as calcium bilirubinate particles by EDS (Fig 4F). Streak like structures (Fig 4G) were confirmed as protein streaks due to the detection of nitrogen by EDS (Fig 4H). Moreover, the EDS percentages for calcium (Fig 4J) and phosphorus confirmed the bulbiform structures seen in Fig 4I are calcium phosphate crystals.

**Table 2:** Elemental composition of a pure cholesterol, mixed cholesterol and a pigment gallstone.

**Element**	**Pure cholesterol GS** *	**Mixed cholesterol GS** *	**Pigment GS** *
**Carbon (C)**	99.51	84.68	23.40
**Oxygen (O)**	00.15	07.31	32.52
**Nitrogen (N)**	00.00	00.94	07.29
**Calcium (Ca)**	00.02	05.99	30.07
**Sodium (Na)**	00.09	00.22	00.69
**Aluminum (Al)**	00.00	00.01	00.04
**Phosphorus (P)**	00.00	00.21	04.68
**Magnesium (Mg)**	00.00	0.017	00.20
**Chlorine (Cl)**	00.06	0.04	00.05
**Manganese (Mn)**	00.00	0.05	01.02
**Cobalt (Co)**	00.00	0.00	00.05

*—Percentage (%) elemental analysis (by weight)

### Pb^2+^ and Cd^2+^ concentrations in GS

Between two toxic heavy metals tested, Pb^**2+**^ was found in all the tested GS samples while Cd^**2+**^ was detected only in 14 (70%) pigment and 3 (21%) cholesterol GS samples. Mean concentrations of Pb^**2+**^ and Cd^**2+**^ in the two types of GS is shown in Table 3. Even though Pb^**2+**^ concentration was significantly higher in pigment GS than cholesterol GS (p = 0.039) a significant difference was not found in the Cd^**2+**^ concentration in two types of GS (p = 0.143).

**Table 3:** Pb^2+^ and Cd^2+^ concentration of cholesterol and pigment gallstone.

**Element**	**Cholesterol GS Mean ± SD**	**Pigment GS Mean ± SD**	**P value** *
**Lead (μg/g)**	18.42 ± 33.80 (*n* = 14)	26.72 ± 23.49 (*n* = 20)	0.039
**Cadmium (μg/g)**	0.16 ± 0.53 (*n* = 14)	0.26 ± 0.39 (*n* = 20)	0.143

*—Two sample t test

## Discussion

Pathogenesis of GS is multifactorial and detection of chemical composition of GS is primarily important to identify their mechanism of formation. To the best of our knowledge this is the first attempt made to identify the aetiopathogenesis of GS among Sri Lankans. Of the three types of GS, pigment and mixed cholesterol stones were the commonest types identified in this Sri Lankan cohort. Further, majority of GS analyzed in this study cohort were composed of multiple chemical compounds namely cholesterol, calcium bilirubinate, calcium carbonate and calcium phosphate. This highlights the importance of exploring the aetiology for calcium salt precipitation in bile in these patients. Presence of calcium bilirubinate in amorphic form indicates the high possibility of precipitation of bilirubinate as polymers.

In the current study, FTIR was used as the basic tool in the analysis of chemical composition of GS as it is an analytical technique with a higher accuracy [13]. One of the major advantages of FTIR in GS analysis is the ability to use only a small amount of sample for testing [13]. The identification of chemical compounds was done by matching wavenumbers obtained for the samples with the standards. The best possible matching was ensured to minimize the errors in the interpretation. Moreover the obtained wavenumbers were cross checked with the existing literature data to improve the accuracy. Absorption peaks at 1465, 1381 and 1056cm^-1^ were taken as fingerprint wavenumbers for cholesterol [12,14,15] while, absorption peaks 1658, 1627, 1566 and 1249 cm^-1^ were used to detect calcium bilirubinate [12,14,15, 20]. Presence of two strong broad peaks at 1080 and 1033cm^-1^, and two small peaks at 609 cm^-1^ and 547 cm^-1^ were used to identify calcium phosphate [12, 21]. Absorption peaks at 1462/1450, 871 and 698/694 cm^-1^ were used to detect calcium carbonate [12, 14, 15] and 2916, 2846, 1543, 1103 and 756 cm^-1^ were taken as the characteristic wavenumbers for calcium palmitate [12, 22].

In the current study, XRD was also used to identify the chemical composition of GS as it is an accurate method to identify the crystalline compounds. XRD results confirmed the presence of all the other chemical compounds identified by FTIR other than calcium bilirubinate. Calcium bilirubinate was not detected by XRD in previous studies [23] and was considered as an amorphous material. This fact was compatible with the findings of the current study. Failure to detect calcium bilirubinate can be recognized as the main reason for infrequent use of XRD to characterize the chemical composition of GS. Being the very first attempt to analyze the composition of GS in Sri Lanka, XRD findings are supportive evidence for FTIR analysis in the present study. Further, SEM and EDS results provided a strong evidence for the occurrence of multiple constituents in the commonest two types of GS identified in this study cohort.

Evidence from the current study indicate that the aetiopathogenesis of GS in our population is likely to be different from that of the Western population, where cholesterol GS are the most predominant [24]. However, common aetiological factors for the development of GS can be anticipated for South Asian region considering the data from other South Asian countries, where majority of GS were mixed or pigment GS [18, 25]. Therefore studying the reasons to have elevated unconjugated bilirubin in bile in majority of South Asian patients with GS is a necessity. Moreover, core of all the mixed cholesterol GS in our study was dark in colour and was composed mainly of calcium salts. Chronic haemolytic anaemia, cirrhrosis, ileal diseases and bacterial infections are the commonly identified reasons for calcium bilirubinate precipitation in bile [1]. However, in addition to generally known aetiological factors, the possible involvement of some other factors on the pathogenesis of pigment GS has also been identified [3, 5, 26]. We have recently described the effect of known aetiological factors on the pathogenesis of mixed cholesterol and black pigment GS using the same study cohort [27]. The study identified obesity as a predicting factor for developing mixed cholesterol GS over black pigment GS. However it failed to detect any significant effect of known environmental risk factors for the development of black pigment GS. Hence it emphasizes the need of further investigations to elucidate the reasons for calcium bilirubinate precipitation in Sri Lankan patients having pigment GS. In this study we identified a significantly high Pb^**2+**^ concentration in pigment GS. However a statistical significance was not identified for Cd^**2+**^ concentration. This might be due to the differences in main excretory routes of these two heavy metals, where Cd^**2+**^ is mainly excreted through urine, while Pb^**2+**^ is excreted through bile [28]. Role of trace metals on the formation of GS is also under evaluation currently [5]. Thus the findings open an important area to be studied in relation to the aetiopathogenesis of pigment GS. Toxic heavy metals at low concentrations induce cytokine mediated inflammatory reactions [29] and toxic heavy metal induced epithelial inflammation [29] can be related to the inflammation of GB as well. Even though this is mainly identified in lungs in smokers [29] similar effects can be expected in biliary tract, which is one of the main pathways of toxic heavy metal excretion [28]. Apart from that, toxic heavy metals have direct effects on calcium signaling system [29]. The toxic heavy metals can penetrate the cell membranes through L—type calcium channels and the intracellular toxic heavy metals in turn cause changes in calcium homeostasis which is identified as a cause of cell death [29]. Due to the competitive inhibition of calcium uptake by calcium channels by toxic heavy metals, the calcium concentration in bile can be increased favoring the formation of calcium bilirubinate [29]. However, further studies are necessary to identify the effects of heavy metals on GB epithelium and their effects on the formation of pigment GS. Moreover, large scale studies to identify the aetiological factors for GS disease in Sri Lanka need to be conducted based on the chemical composition of GS as revealed by the current study.

## Conclusion

GS disease in Kandy district, Sri Lanka is common among middle aged females and multiple chemical compounds are involved in GS formation. Presence of different calcium salts (calcium bilirubinate, calcium carbonate and calcium phosphate) is a common feature. Majority of the GS were either pigment or mixed cholesterol with a pigment nidus, denoting the possible role of elevated unconjugated bilirubin in bile on the pathogenesis of GS. Hence it is imperative to explore this further, and it would specially be helpful in understanding the aetiopathogenesis of GS among Sri Lankans.
